# Transport Properties of Solutions in γ–FeOOH/CSH Pores of Steel Fiber-Reinforced Concrete (SFRC) Derived Using Molecular Dynamics

**DOI:** 10.3390/ma18102176

**Published:** 2025-05-08

**Authors:** Yalin Luan, Runan Wang, Changxin Huang, Andrey Jivkov, Lianzhen Zhang

**Affiliations:** 1 College of Pipeline and Civil Engineering, China University of Petroleum, Qingdao 266580, China; luanyalin@upc.edu.cn (Y.L.); 19862857721@163.com (R.W.); 2 School of Civil Engineering, Shandong University, Jinan 250014, China; 202320792@mail.sdu.edu.cn; 3 School of Engineering, The University of Manchester, Manchester M13 9PL, UK; andrey.jivkov@manchester.ac.uk

**Keywords:** steel fiber-reinforced concrete, molecular dynamics simulations, γ–FeOOH/CSH, transport behavior, atomic density distribution

## Abstract

Steel fiber-reinforced concrete structures designed for marine environments can become compromised by the ingress of water and ions. Water and ion transport through the pores between steel fibers and concrete gels significantly affects the durability of such structures, but the mechanisms of this transport are not sufficiently understood. Reported here is a molecular dynamics-based investigation of the transport of water, NaCl, Na_2_SO_4_, and mixed solutions of NaCl and Na_2_SO_4_ through γ–FeOOH/CSH pores. The effect of pore width on the capillary transport of NaCl + Na_2_SO_4_ solutions was also investigated and reported. It is shown that the depth of water penetration in NaCl solution increases parabolically with time. It is further shown that the CSH surface forms bonds with different ions to form Na–O_CSH_, Cl–Ca_CSH_, and S–Ca_CSH_ compounds, which results in reduced rates of solution transport. The mixed NaCl + Na_2_SO_4_ solution was found to have the lowest transport rate. A reduction in pore width was found to reduce the transport rate of water molecules and diminish the transport of ions. In pores smaller than 2.5 nm in width, the immobilized ions aggregate into clusters, occupying pore inlets and blocking more ions from entering the channels. Compared with the matrix on both sides, solutions are transported significantly faster along the CSH side than along the γ–FeOOH side, indicating that the addition of steel fibers can effectively slow down the transport of water molecules and ions in concrete. These data on the difference in the transport of solutions along the two sides of the matrix may provide molecular-level insights to support studies on the durability of concrete materials.

## 1. Introduction

Improving the durability of concrete structures will contribute substantially to the sustainable development goals of modern societies [1]. This is a challenging problem requiring urgent solutions [2]. One approach involves the incorporation of steel fibers to produce steel fiber-reinforced concrete (SFRC) [3]. This has become widespread in engineering applications [4], particularly in materials for use in marine environments [5]. The number of port construction projects has been increasing year by year. In China alone, the ocean engineering manufacturing industry generated an added value of CNY 103.2 billion by 2024, an increase of 9.1% over the previous year [6]. Compared to conventional concrete, SFRC composites have superior strength [7,8], crack resistance [9,10], and load–bearing capacity, making them excellent structural materials [11]. However, the interfaces between concrete and steel fibers are weak points in the composite microstructure [12,13] and significantly impact the durability of concrete materials [14]. First, they are pathways for the faster transport of water and ions compared to the rest of the concrete body. Second, they are mechanically weaker and are further weakened by erosion/corrosion by water and ions, making them the likely locations for mechanical damage and crack initiation [15]. Third, the appearance of cracking patterns in SFRC during failure [16] has a strong impact on the nanomechanical properties of local specimen regions [17].

The detrimental effects of water and ion ingress on the mechanical behavior and interfacial properties of SFRC have been investigated via macroscopic-scale experiments [18,19,20]. Hwang et al. [18] employed an electrically accelerated method to examine the corrosion at the interface between steel fibers and concrete matrices. Their findings showed that the porosity of the interface is higher than the porosity of the matrix and that this porosity facilitates chloride ion transport and leads to steel fiber corrosion. Their conclusion is that SFRC is at higher risk of corrosion than is classical reinforced concrete. Yoo et al. [19] studied the corrosion of SFRC exposed to a 3.5% chloride salt solution and reported a direct correlation between the degree of fiber corrosion and surface roughness. Their results show that when the steel loss due to corrosion ranges from 4% to 6%, the tensile properties of SFRC improve. However, exceeding this threshold results in fiber breakage and the subsequent deterioration of concrete performance. Tai et al. [20] compared the performance degradation of SFRC subjected to chloride erosion to that of SFRC subjected to combined carbonation and chloride erosion. Their results show that corrosion progresses faster under the coupled effect of carbonation and chloride erosion. After 360 wet–dry–wet cycles of chloride exposure, steel fibers within approximately 10 mm of the surface were severely corroded, while deeper fibers remained unaffected despite chloride ion penetration exceeding 40 mm.

The macroscopic experimental results show that water and ions gradually infiltrate SFRC [21] and cause structural damage [22]. However, the exact mechanisms of the transport of water and ions within pores cannot be fully elucidated through macroscopic experiments alone. A better understanding can be obtained by the investigation of transport at the microscopic level, specifically focusing on the steel fiber/concrete interface. Molecular dynamics (MD) simulations have been used to study the structure [23], kinetics [24], and interfacial behavior [25,26] of harmful ions in gel pores, providing insights into the mechanisms of transport of water and ions. Calcium silicate hydrate (CSH) [27], formed by the hydration of cement clinker [28], is the primary component of concrete-based materials [29]. Hou et al. [25] simulated the transport of NaCl, Na_2_SO_4_, and mixed NaCl + Na_2_SO_4_ solutions in CSH gel pores. Their finding was that different ions interact with sulfate ions during transport, inhibiting the movement of chloride ions. Large Na–SO_4_ clusters block nanochannels, slowing the entry of water into gel pores. Yang et al. [26] simulated the capillary transport of chloride salts in the CSH gel pores of varying sizes. Their findings were that reduced pore size affects water–ion bonding and that channel narrowing inhibits penetration by larger ions. These studies provide valuable microscopic-level information and a theoretical foundation for understanding the intrusion of water and ions into CSH gel pores. However, the transport and bonding mechanisms of water and ions in the pores formed between concrete and steel fibers in SFRC have not been investigated to date.

The present work aimed to better understand the transport behavior and microscopic mechanisms of water and ions within the pores of SFRC. The investigation was performed by molecular dynamics. Initially, a transport model was developed for water, NaCl solution, Na_2_SO_4_ solution, and a composite NaCl + Na_2_SO_4_ solution within the pores of SFRC. The differences in transport characteristics across these solution environments were examined. Subsequently, four distinct pore widths (3.5 nm, 2.5 nm, 1.5 nm, and 1 nm) were considered to analyze the transport process of the NaCl + Na_2_SO_4_ composite solution. Through the analysis of local structures and intrusion behaviors, the results elucidated the migration mechanisms of water and ions within the pores of SFRC under varying solution types and pore dimensions. These findings provide valuable insights into the degradation mechanisms of SFRC in marine environments.

This article was organized as follows: In Section 2, we provide a detailed introduction to the modeling and simulation processes based on MD. In Section 3, the simulation results are analyzed and discussed. First, the transport process was demonstrated by snapshots, and the invasion depth differences in different solutions in the pores were compared. Second, by calculating the density distribution and RDF of each particle, the bonding differences between different solutions and matrices were revealed. Third, the transport process of NaCl + Na_2_SO_4_ mixed solution at different pore sizes was analyzed. Fourth, the influence mechanism of different pore sizes on solution transport was studied. Finally, the conclusion is followed up in Section 4.

## 2. Experiment and Model

### 2.1. Experiment

Steel fiber-reinforced concrete (SFRC) specimens with dimensions of 100 mm × 100 mm × 100 mm were prepared using steel molds. The concrete mix proportions are listed in Table 1 [30]. After casting, the specimens were stored indoors at a temperature of 293 K for 48 h before demolding, followed by standard curing for 28 days to ensure proper hydration and strength development.

**Figure 1:**
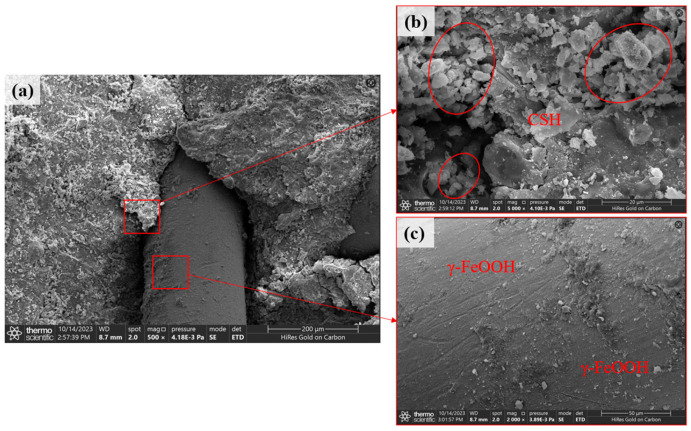
SEM imaging of (**a**) the γ–FeOOH/CSH interface at 500× magnification, (**b**) CSH at 5000× magnification, and (**c**) γ–FeOOH at 2000× magnification.

After curing, the specimens were subjected to uniaxial compression at a uniform loading rate until failure. To investigate the interfacial microstructure, the steel fiber-containing samples were extracted from the surface layer (within 5 mm depth) of the fractured specimens. These samples were then immersed in anhydrous ethanol to terminate further hydration and subsequently dried in an oven for 24 h until a constant weight was achieved. The dried samples were examined using a JSM–6490LV scanning electron microscope (SEM) to characterize their microstructural features. Key observations from the SEM analysis are shown in Figure 1.

SEM imaging at 500× magnification, shown in Figure 1a, revealed the presence of abundant CSH, which served as the primary binding phase in cementitious materials. Higher-magnification image at 5000× in Figure 1b showed that the CSH exhibited diverse morphologies, including fibrous, flocculent, granular, and densely packed structures. These microstructural features effectively interconnected cement particles and hydration products, contributing to the overall compactness of the matrix. A rough passivation layer was observed in an image at 2000× in Figure 1c, indicating possible surface oxidation or chemical interaction between the steel fibers and the surrounding cementitious matrix.

The region between the steel fibers and the concrete matrix, known as the interfacial transition zone (ITZ), exhibited a looser microstructure with higher porosity and fewer hydration products compared to the bulk matrix. This structural heterogeneity resulted in reduced bond strength and increased permeability, making the ITZ a critical weak point for the ingress of aggressive solutions.

While SEM provided valuable insights into the microstructural characteristics of SFRC, its resolution and method limited the ability to analyze solution transport mechanisms within micropores. To address this gap, an MD model was built to better understand the penetration behavior of corrosive media in the ITZ and optimize the durability performance of SFRC.

### 2.2. Model Construction

**Figure 2:**
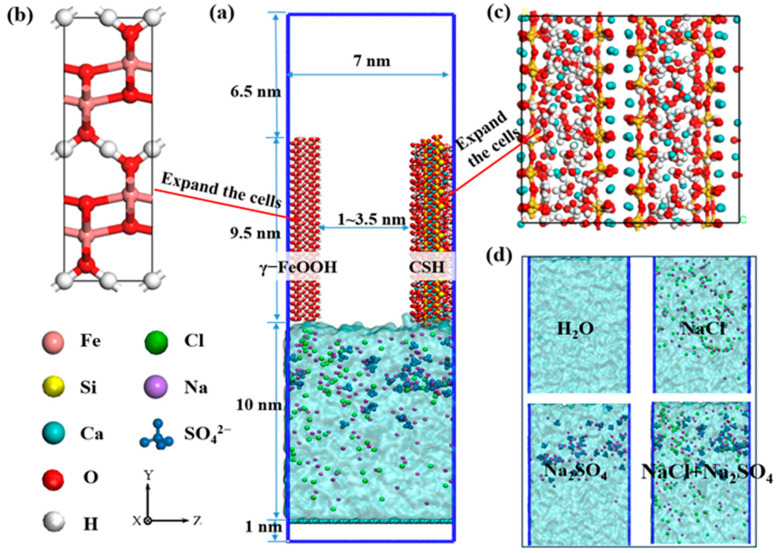
(**a**) Model setup for solution transport through γ–FeOOH/CSH channel; (**b**) structure of γ–FeOOH; (**c**) structure of CSH; (**d**) solution cases: H_2_O, NaCl solution, Na_2_SO_4_ solution, and mixed NaCl + Na_2_SO_4_ solution.

The transport model consisted of a pore channel of approximately 9.5 nm width in contact with the environment of different solutions at the bottom. A fraction of the cement hydration product was calcium hydroxide, which made the concrete a strongly alkaline environment [31]. In such environments, a passive film was formed on the surface of the steel fibers [32,33]. The main component of the passive film was γ–FeOOH [34]. Figure 2a shows the pore channel model with one wall representing the steel fiber passivation film (γ–FeOOH) and the other wall the calcium silicate hydrate (CSH). The interlayer distances of 1 nm, 1.5 nm, 2.5 nm, and 3.5 nm are chosen by the following SEM-derived measurements of the interfacial pores between steel fibers and the CSH matrix and supported by previous simulations of comparable systems [26]. The passive film wall was constructed by cleaving the bulk crystal of γ–FeOOH along the [0 1 0] direction as shown in Figure 2b [35]. The crystal structure of γ–FeOOH was expanded in every direction by corresponding multiples, and the γ–FeOOH supercell model with dimensions 22.6 Å × 95 Å ×15 Å was built. For the creation of the CSH wall, the initial crystal structure was tobermorite with Ca/Si = 1.7, as depicted in Figure 2c. A realistic hydration product model was built by removing the H_2_O molecules and randomly deleting some of the bridging Si–O tetrahedra in the silicon chains [36,37]. Thus, the CSH supercell model with dimensions 22.6 Å × 95 Å ×20 Å was constructed. To elucidate the fundamental mechanisms of solution transport within nanopores, simplified models were employed without considering the roughness and heterogeneity of the interfaces [38]. Four different water solutions were selected as shown in Figure 2d: pure water, 5% NaCl solution, 5% Na_2_SO_4_ solution, and 5% NaCl + 5% Na_2_SO_4_ solution, which approximated the actual seawater environment [39]. The real environment of seawater, such as the marine pH, is also influenced by various factors such as temperature, salinity, atmospheric CO_2_ partial pressure, and biological activities. However, the influences of the concentration changes of Ca^2+^ ions, Cl^−^ ions, and SO_4_^2−^ ions on the overall pH value of seawater were relatively small. The number of atoms and ions in the four solutions is given in Table 2. A graphene sheet was placed at the bottom of the solutions to set a rigid body condition.

### 2.3. Force Field and Molecular Dynamics Procedure

Molecular dynamics (MD) simulations of the model were carried out using the Large-scale Atomic/Molecular Massively Parallel Simulator 8Feb2023 (LAMMPS 8Feb2023) [40] free software package. The visualization of the processes was realized in Visual Molecular Dynamics (VMD) [41]. The ClayFF force field [42] was used in the simulation due to its general applicability for simulating various cement hydration products, interfaces between minerals and solutions, adsorption of anions and cations on hydroxide surfaces, and its success in simulating the interatomic potentials between the atoms in the solution and the calcium silicate calcium skeleton.

**Figure 3:**
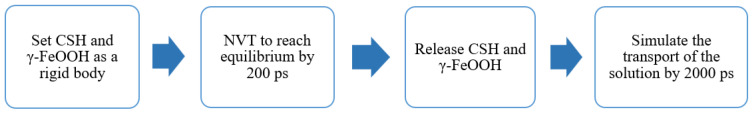
Flowchart of the simulation process.

The truncation distances for van der Waals (vdW) and short-range electrostatic interactions were set at 10 Å, whereas the long-range electrostatic interactions were calculated using the Particle–Particle–Particle–Mesh (PPPM) method [43]. The simulations were performed as follows: First, both the CSH and γ–FeOOH walls were set as rigid bodies, i.e., a “frozen” state. This setting avoided simulation errors caused by the deformation of nanopores, enabling the simulation to focus more on the interaction between fluid molecules and nanopore walls [44]. An invisible wall was placed between the entrance of the pore channel and the bottom solution to prevent water molecules and ions from entering the pore. Second, the simulation was performed under NVT ensemble at 300 K via the Nose–Hoover thermostat, and the solution was relaxed for 200 ps to reach equilibrium [35,45]. Third, the “frozen” walls were released, and the simulation was performed under the NVT ensemble to achieve the equilibrium state of the substrates and the surface groups. Finally, the invisible wall at the entrance of the pore channel was removed to allow water and ions to enter freely through the channel under the NVT ensemble for 2000 ps. The flowchart of the simulation process is shown in Figure 3. During the simulation, the atomic trajectories from 0 to 2000 ps were detected and recorded with an interval of every 10 fs with a time step of 1 fs [46]. Periodic boundary conditions were applied in all directions to eliminate the size effect, simulating an infinite system and making the simulation results more accurate, reliable, and closer to the behavior of real physical systems [47].

## 3. Results and Discussion

### 3.1. Water and Ions Transport in the γ–FeOOH/CSH Pore

**Figure 4:**
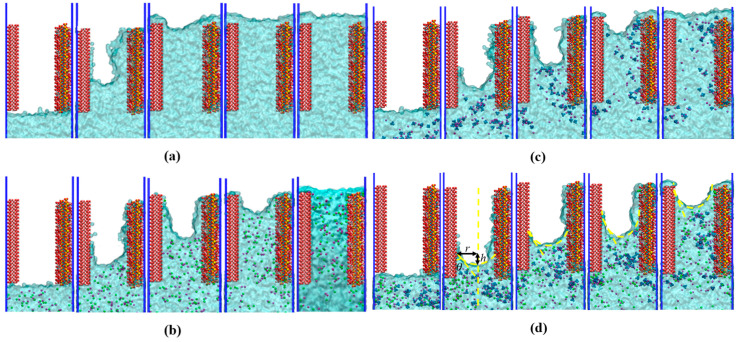
Snapshots of capillary flow of water and ions in 3.5 nm pore at *t* = 0, 100, 600,1000, and 2000 ps in (**a**) water; (**b**) NaCl; (**c**) Na_2_SO_4_; (**d**) NaCl + Na_2_SO_4_. (Color codes for atoms were pink: Fe; yellow: Si; cyan: Ca; red: O; white: H; green: Cl; purple: Na; blue: SO_4_^2−^).

Figure 4 shows the transport of the four solutions at different times. It can be seen that the solution produced capillary adsorption in all four models. Among them, the water molecules were the fastest, driving the Na^+^ ions, Cl^−^ ions, and SO_4_^2−^ ions forward in the pore. The water and ions gradually filled the entire pore of γ–FeOOH/CSH within 2000 ps. The contact angles of the NaCl + Na_2_SO_4_ mixed solution on the solid surfaces were calculated to examine the morphology of the interfacial meniscus. The contact angle *θ* was calculated by Formula (1):

(1) $$\theta =\tan^{-1}\left(\frac{r^{2}-h^{2}}{2rh}\right)$$

where *r* is the radius of the nanopore, and *h* is the height of the meniscus, as shown in Figure 4. The contact angles between the solution and both interfaces were less than 90°, confirming the hydrophilicity of both γ–FeOOH and CSH. The transport of water and ions was significantly faster on the surface of CSH than on the surface of γ–FeOOH. At *t* = 100 ps, the CSH surface was completely covered by water molecules, while only 30% of the γ–FeOOH surface was covered with water. This confirmed that CSH was more hydrophilic than γ–FeOOH and had a greater adsorption effect on water molecules. During the transport in the mixed solution shown in Figure 4d, the contact angles on the surface of γ–FeOOH gradually decreased from 44.7°, 38.1°, 31.5° to 27.6° over time, while the contact angles on the surface of CSH gradually decreased from 39.7°, 35.8°, 38.3° to 23.7°. The reduction in the contact angles as the solution gradually migrated into the γ–FeOOH/CSH pore conformed to the capillary transport law of water and ions [48].

Figure 4a–d show that the water molecules migrate the fastest in a pure water environment compared with the solutions containing ions. At *t* = 600 ps in a water environment, the water molecules had filled the pore, and the meniscus-like shape between the solution and the pore matrix had disappeared. In contrast, at *t* = 600 ps in the other solutions, the water molecules migrated between 1/2 and 2/3 of the entire pore. It can be inferred that the addition of ions slowed down the capillary transport rate of the water molecules. It can be further seen in Figure 4b,c that the water and ions migrated slightly faster in the NaCl solution than in the Na_2_SO_4_ solution. At *t* = 100 ps, the water molecules in the Na_2_SO_4_ solution had migrated to about 25% of the entire pore on the γ–FeOOH wall, while in the NaCl solution, they had migrated to nearly 35%. The reason for the slower ingress of the water and ions in the Na_2_SO_4_ solution was mainly due to the aggregation of Na^+^ ions and large SO_4_^2−^ ions on the CSH surface as shown in Figure 4c. Furthermore, the aggregation of the Na^+^ ions and large SO_4_^2−^ ions into clusters at the entrance of the nanopore hindered the migration of the water and ions. The figure also shows that the transport of the water and ions was the slowest in the mixed NaCl + Na_2_SO_4_ environment. This was due to enhanced ion absorption on the CSH wall. Different types of ions, including the Na^+^ ions, Cl^−^ ions, and SO_4_^2−^ ions, agglomerated and blocked the solution flow.

**Figure 5:**
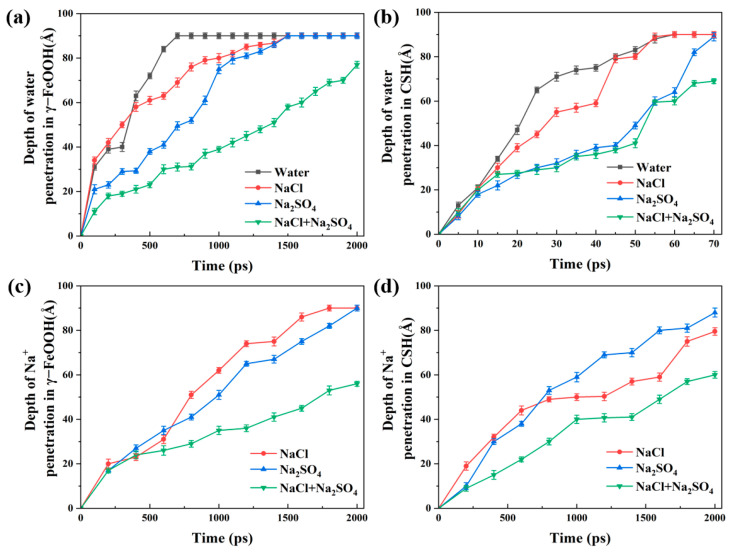

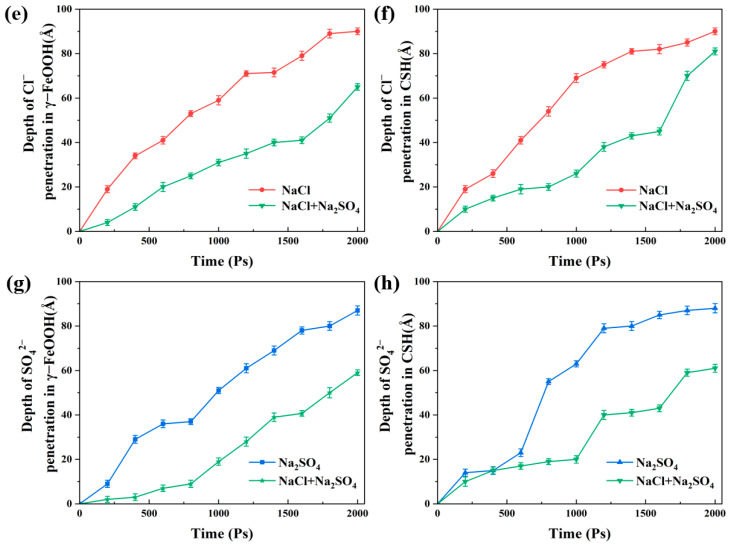
Penetration depths of (**a**) water, (**c**) Na^+^, (**e**) Cl^−^, and (**g**) SO_4_^2−^ along the γ–FeOOH surface and penetration depths of (**b**) water, (**d**) Na^+^, (**f**) Cl^−^, and (**h**) SO_4_^2−^ along the CSH surface under different solutions.

The capillary transport of solutions in the γ–FeOOH/CSH pore was quantified by recording the penetration depths of water molecules and ions along both pore surfaces and was presented in Figure 5. By comparing Figure 5a,b, it was observed that the surface of CSH was fully covered by the water molecules at *t* = 70 ps, whereas the surface of γ–FeOOH was fully covered at *t* = 1500 ps. This clearly showed that the water molecules traveled much faster on the CSH surface than on the γ–FeOOH surface. The penetration depths of water molecules in different solutions at any given time can be ordered as follows: water > NaCl > Na_2_SO_4_ > NaCl + Na_2_SO_4_. In addition, the penetration depths of water molecules in the NaCl solution on both the γ–FeOOH and CSH surfaces exhibited approximately parabolic dependence on time, which is consistent with the Lucas–Washburn equation in the capillary adsorption theory [49].

The penetration depths of the Na^+^ ions, Cl^−^ ions, and SO_4_^2−^ ions in different solutions on both sides of the pore are depicted in Figure 5c–h. The penetration depth of the ions in the mixed NaCl + Na_2_SO_4_ solution was smaller than that in the NaCl or Na_2_SO_4_ solution. At *t* = 2000 ps, the penetration depth of the Na^+^ ions reached 90 Å in the NaCl or Na_2_SO_4_ solution on both sides of the pore, which was much greater than that of about 60 Å in the mixed solution of NaCl + Na_2_SO_4_. The transport rate of the Na^+^ ions was significantly slower in the mixed solution than in the NaCl or Na_2_SO_4_ solution. The transport rate was obtained as the ratio of the distance increment to the time increment, which is the slope of the curve. It can be derived from Figure 5c,d that Na^+^ ions were significantly slower in the mixed solution than in the NaCl or Na_2_SO_4_ solution. An analysis of the instantaneous transport rates revealed a consistent temporal trend characterized by an initially elevated rate followed by subsequent attenuation. The maximum observed transport rates in NaCl and Na_2_SO_4_ solutions reached 0.095 Å/ps and 0.1 Å/ps, respectively, while the minimum rates decreased to 0.0015 Å/ps and 0.005 Å/ps. The mean transport rates were calculated as 0.044 Å/ps for NaCl and 0.04 Å/ps for Na_2_SO_4_ solution. In contrast, the mixed solution of NaCl + Na_2_SO_4_ exhibits a maximum rate of 0.05 Å/ps, a minimum rate of 0.0035 Å/ps, and an average rate of 0.03 Å/ps. Notably, the single-component solutions demonstrate significantly faster mean transport rates compared to the mixed solution, with an average rate enhancement of 233.3%. The same was observed for Cl^−^ and SO_4_^2−^ ions. These results indicated that the coupling of these ions slows down the transport of water and ions. On the surfaces of γ–FeOOH and CSH, the time required for the ions to pass through the 9 nm nanopore was always longer than for the water molecules. In the NaCl or Na_2_SO_4_ solution environment, ions could completely pass through the pore within 2000 ps. This confirmed that the ion transport rate was lower than the water molecule transport rate and that the ions were carried forward by water molecules.

**Figure 6:**
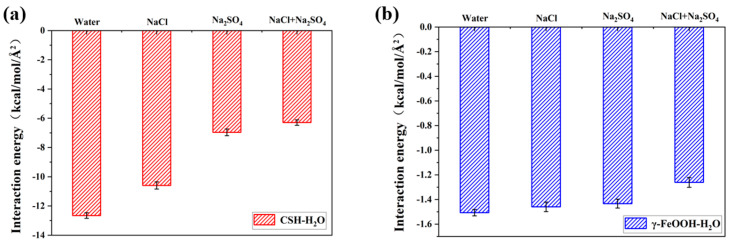
Interaction energy per unit contact area of (**a**) CSH–H_2_O and (**b**) γ–FeOOH–H_2_O.

From the water penetration depth perspective, there was no clear difference between the surfaces of the γ–FeOOH and CSH substrates. To clarify the adsorption difference in the water molecules on the substrates, the interaction energy per unit area between each substrate and the water molecules was calculated by Formula (2):

(2) $$E_{1/2}=\frac{E_{total}-(E_{1}+E_{2})}{A_{1/2}}$$

where *A*_1/2_ is the cross-sectional area of components 1 and 2; *E*_1/2_ represents the interaction energy per unit area between components 1 and 2; *E*_total_ is the total energy of the system containing components 1 and 2; and *E*_1_ and *E*_2_ are the energies of components 1 and 2, respectively. The calculated interaction energies are shown in Figure 6. It can be seen that the interaction energy between the substrates and the water molecules can be ordered as follows: water > NaCl > Na_2_SO_4_ > NaCl + Na_2_SO_4_. This is consistent with the order of transport rates of water shown in Figure 4. *E*_CSH–H2O_ = −12.66 kcal/mol/Å^2^ in a water environment was 50.3% lower than *E*_CSH–H2O_ −6.3 kcal/mol/Å^2^ in NaCl + Na_2_SO_4_ solution. In these two different environments, *E*_γ–FeOOH–H2O_ decreased from −1.5 kcal/mol/Å^2^ to −1.26 kcal/mol/Å^2^, i.e., by 16.7%. This showed that the high transport rate of the water molecules in the nanopore was due to their mutual attraction with the substrates. Considering that *E*_CSH–H2O_ was much larger than *E*_γ–FeOOH–H2O_, and that the water molecules migrated faster on the surface of CSH than on the surface of γ–FeOOH, it can be inferred that the higher the interaction energy, the higher the transport rate. The addition of ions decreased the interaction energy between the substrates and the water molecules, leading to a reduction in the transport rate. In addition, the γ–FeOOH substrate slowed the water transport, thereby inhibiting the corrosion of the steel fibers.

### 3.2. Local Structure of Different Solutions in the γ–FeOOH/CSH Pore

**Figure 7:**
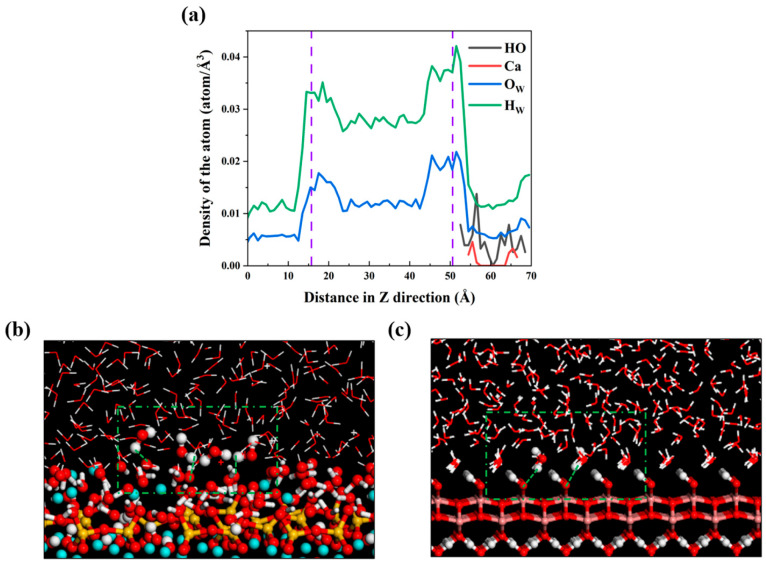
(**a**) Atomic density distribution of H_w_ and O_w_ across the γ–FeOOH/CSH channel, and snapshots of the water molecules on the surface of (**b**) CSH and (**c**) γ–FeOOH. (Color codes for atoms were pink: Fe; yellow: Si; cyan: Ca; red: O; white: H; green: Cl; purple: Na; blue: SO_4_^2−^).

The distribution of the water molecules and ions on the interfaces was investigated by an analysis of the density distributions. Figure 7a shows the density distribution of water molecules in the nanopore in the Z direction. Multiple peaks of H_w_ and O_w_ (H and O atoms in water molecules) were present on both surfaces of the γ–FeOOH and CSH substrates. On the CSH side, H_w_ had four peaks at Z = 45.5, 49.5, 51.5, and 52.5 Å. The highest density of H_w_ was 0.042 atoms/Å^3^, found at the third peak, Z = 51.5 Å. On the γ–FeOOH side, H_w_ had also four peaks at Z = 14.5, 16.5, 18.5, and 20.5 Å. The highest density of H_w_ was 0.035 atoms/Å^3^, found at the third peak, Z = 18.5 Å. The distribution of O_w_ showed a similar trend. Multiple peaks in the density of these elements on the pore surfaces implied a layered accumulation of water molecules [50,51]. H_w_ was denser than O_w_ at the surfaces, indicating that the hydrogen atoms in water molecules occupied more sites on the surface of γ–FeOOH and CSH, forming hydrogen bond linkages. This confirmed the hydrophilicity of the two substrates. Compared with γ–FeOOH, the peak densities of H_w_ and O_w_ were larger and closer to the CSH surface. The results indicate that CSH has stronger hydrophilicity. Snapshots of water molecules at the substrate surfaces are shown in Figure 7b,c. It can be seen that H_w_ formed hydrogen bonds with O atoms exposed on the surface of the substrate. H_w_ points to the substrate, which affected the orientation of water molecules by the formation of hydrogen bonds.

**Figure 8:**
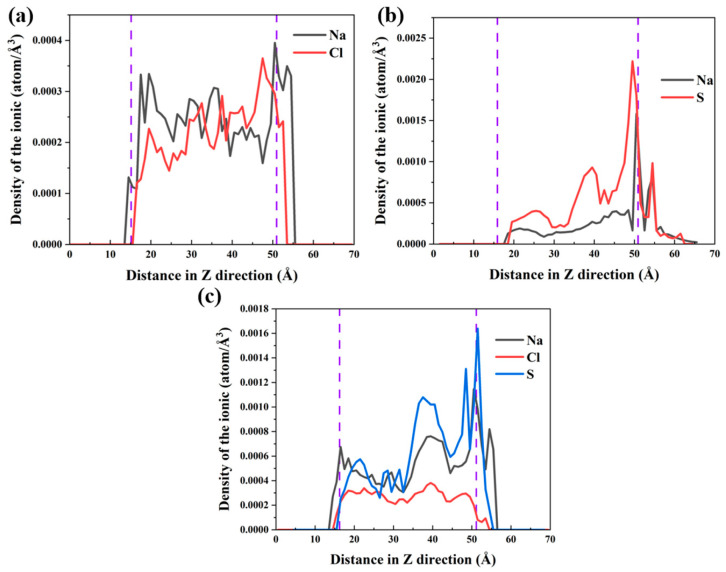
Atomic density distributions of ions across γ–FeOOH/CSH channel: (**a**) NaCl (Na, Cl), (**b**) Na_2_SO_4_ (Na, SO_4_^2−^), and (**c**) NaCl + Na_2_SO_4_ (Na, Cl, SO_4_^2−^). (The left dashed line in the figure represents the surface of γ–FeOOH, while the right dashed line represents the surface of CSH).

**Figure 9:**
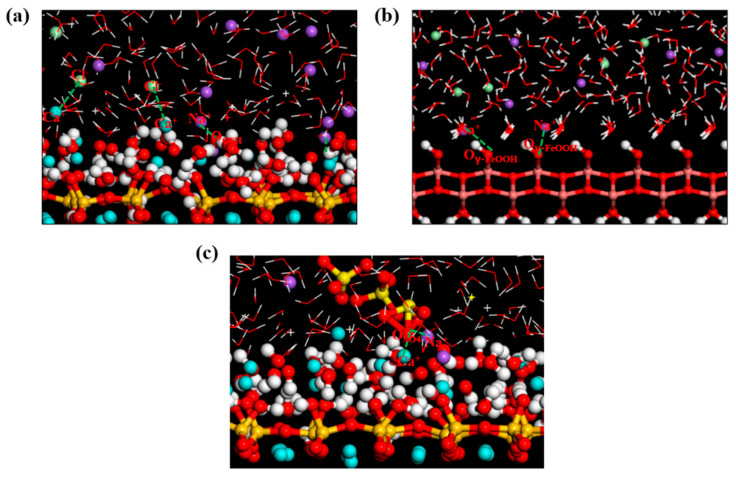
Snapshots of ions in NaCl solution on (**a**) CSH and (**b**) γ–FeOOH surfaces, and (**c**) snapshots of ions in Na_2_SO_4_ solution on the surface of CSH. (Color codes for atoms were, pink: Fe; yellow: Si; cyan: Ca; red: O; white: H; green: Cl; purple: Na; blue: SO_4_^2−^).

The density distribution of ions in the studied solutions is shown in Figure 8. It can be seen in Figure 8a that distinct peaks appeared in the density distribution of both the Na^+^ and Cl^−^ ions in the NaCl solution. Compared to the peaks of the Cl^−^ ions, the peaks of the Na^+^ ions were higher and narrower. The distance between the Na^+^ ions and the CSH surface was 0.5 Å, while the distance between the Cl^−^ ions and the CSH surface was 2.5 Å. Similar trends were observed on the surface of γ–FeOOH: Na^+^ was closer to γ–FeOOH than Cl^−^, which indicates that the γ–FeOOH and CSH surfaces were attractive to cations and repulsive to anions. The Na^+^ ions were denser on the CSH surface than on the γ–FeOOH surface. The adsorption of the Cl^−^ ions was different, and a larger density distribution peak appeared only on the surface of CSH. From the snapshots of ions on the surfaces of the substrates shown in Figure 9a,b, it could be seen that the Na^+^ ions bind to the hydroxyl oxygen atoms and aggregate on the surfaces of γ–FeOOH and CSH, while the Cl^−^ ions bonded with the Ca^2+^ ions in CSH [52].

It can be seen in Figure 8b that the Na^+^ and SO_4_^2−^ ions in the Na_2_SO_4_ solution were concentrated at the surface of CSH. The density distribution of both the Na^+^ and SO_4_^2−^ ions had sharp peaks, different from the wide peaks of the Cl^−^ ions, indicating that CSH had a strong adsorption effect on these ions. The maximum density of the Na^+^ ions was at 1 Å from the surface of the substrate, and that of the SO_4_^2−^ ions was at 2 Å from the substrate, indicating that the Na^+^ ions were adsorbed more strongly than the SO_4_^2−^ ions. Compared to the NaCl solution, Na^+^ ions in the Na_2_SO_4_ solution tended to aggregate mostly on the CSH surface, indicating that the adsorption of the SO_4_^2−^ ions affected the distribution of the Na^+^ ions. From the snapshots in Figure 9c, it was observed that a Ca–SO_4_–Na bond was formed after the oxygen atoms in the SO_4_^2−^ ions bonded with the Ca^2+^ ions and adsorbed the Na^+^ ions on the surface of CSH. This inhibited the aggregation of the Na^+^ ions on the γ–FeOOH surface.

The density distribution of ions in a mixed solution of NaCl + Na_2_SO_4_, given in Figure 8c, showed several peaks of Na^+^ ions on both the γ–FeOOH and CSH surfaces as well as several peaks of SO_4_^2−^ ions on the CSH surface. This implied that CSH strongly attracts Na^+^ ions and SO_4_^2−^ ions under different ion coupling. On the contrary, there were no significant density peaks of Cl^−^ ions on the CSH surface, indicating that the addition of sulfate weakened the interaction between CSH and Cl^−^ ions. As the sites of the Ca^2+^ ions on the CSH surface were occupied by the SO_4_^2−^ ions, the binding of the Cl^−^ ions to the Ca^2+^ ions was inhibited, which promotes the stripping of Cl^−^ from the substrate.

**Figure 10:**
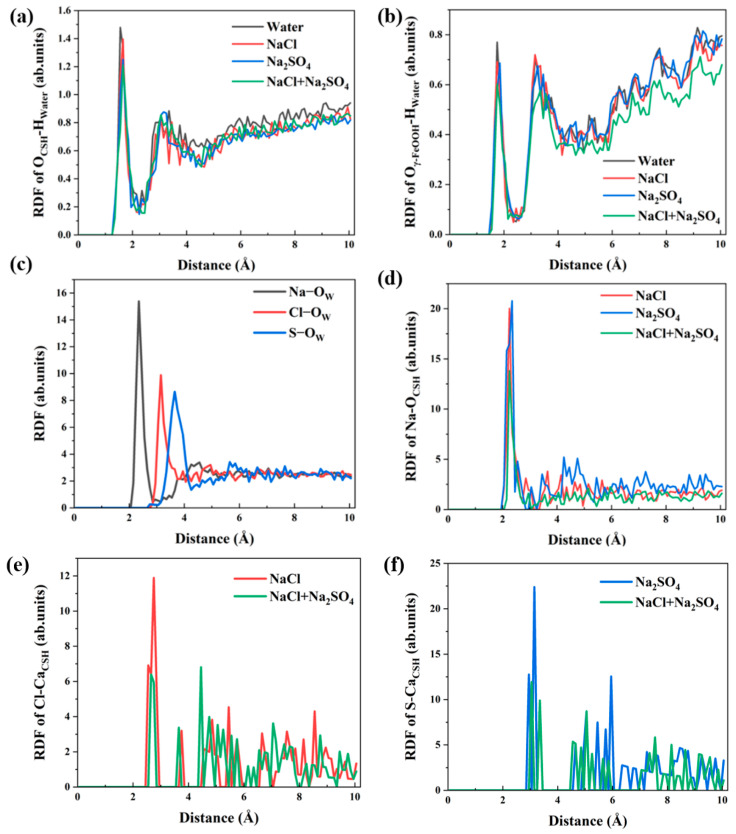
RDF of (**a**) O_CSH_–H_Water_, (**b**) O_γ–FeOOH_– H_Water_, (**c**) Na, Cl, S–O_W_, (**d**) Na– O_CSH_, (**e**) Cl–Ca_CSH_, and (**f**) S–Ca_CSH_.

To further understand the effect of the chemical environment on the transport in γ–FeOOH/CSH pores and the interactions between the environmental components, the radial density function (RDF) was calculated, and the results are shown in Figure 10. Figure 10a,b show the RDF curves of O_CSH_–H_Water_ and O_γ–FeOOH_–H_Water_. The first peaks of the two curves were at 1.75 Å and 1.95 Å, respectively, which were less than the distance required for the formation of hydrogen bonds between oxygen and hydrogen (2.45 Å). This indicated that the transport of water molecules was promoted by the hydrogen bonds formed between the two substrates and the water molecules in the solutions. Comparing the RDFs of all solutions, the peaks were lowest in the NaCl + Na_2_SO_4_ solution, indicating that the hydrogen bonds in this solution were the weakest, resulting in the slowest migration rate of water molecules. The RDF peak of O_CSH_–H_w_ was significantly higher than that of O_γ–FeOOH_–H_w_ in the same solution. Taking NaCl + Na_2_SO_4_ as an example, the RDF peak of O_CSH_–H_w_ was 1.21, twice the peak of O_γ–FeOOH_–H_w_, which was 0.6. This implied that the surface of CSH was more likely to form hydrogen bonds with water molecules. Therefore, the transport rate of water molecules on the surface of CSH was much faster than that on the surface of γ–FeOOH.

As shown in Figure 10c, there were RDF peaks of Na–Ow, Cl–O_W,_ and S–Ow at 2.25 Å, 3.15 Å, and 3.65 Å, respectively. This indicated that the Na^+^ ions, Cl^−^ ions, and sulfates interacted strongly with the surrounding water molecules and were prone to aggregate. The position of the RDF peak represented the optimal radius between the ion and the O_Water_ atom. The RDF peak of Na–O_w_ was the first to appear at 2.35 Å and was the largest. This showed that the interaction between the Na^+^ ions and water molecules was the strongest, and the distribution distance was the shortest.

The RDF of Na–O_CSH_ presented in Figure 10d had a first peak at 2.25 Å, indicating that the hydroxyl oxygen on the CSH surface attracted positively charged Na^+^ ions, and the Na–O_CSH_ bond length was 2.25 Å. Figure 10e, f depict the RDFs of Cl–Ca_CSH_ and S–Ca_CSH_, respectively. The first peaks of the two curves were at 2.95 Å and 3.15 Å, respectively, implying the Cl^−^ ions and SO_4_^2−^ ions had significant spatial correlations with the Ca^2+^ ions exposed on the CSH surface so that Cl–Ca and SO_4_–Ca pairs were easily formed. Compared with the single-salt solutions, the RDF peaks of Cl–Ca_CSH_ and S–Ca_CSH_ in the mixed solution were substantially lower, which indicated that the Cl^−^ ions and SO_4_^2−^ ions occupied the binding sites of the Ca^2+^ ions on the CSH surface in the mixed solution, leading to a smaller number of Cl–Ca and SO_4_–Ca ion pairs. The effect of multiple ions was that the CSH interface and ions were prone to form large cluster ion pairs such as O_CSH_–Na–SO_4_, O_CSH_–Na–Cl, SO_4_–Ca–Cl–Ca, etc., which was consistent with the observation in Figure 4c,d. The formation of large clusters of ion pairs blocked the transport of water and ions through the gel pores to some extent. In contrast, the exposed part of γ–FeOOH was a hydroxyl group, which had an adsorption effect on the Na^+^ ions. Although Na–O_γ–FeOOH_ was easy to form, it was difficult to form Cl–O_γ–FeOOH_ and SO_4_–O_γ–FeOOH_. As a result, it was more difficult to form a large cluster of ion pairs on the γ–FeOOH surface, and the adsorption of the Cl^−^ ions and SO_4_^2−^ ions was weak.

### 3.3. Water and Ions Transport in Different Pore Widths

The results presented in the previous subsections showed that the rate of transport in the γ–FeOOH/CSH pore was the smallest for the NaCl + Na_2_SO_4_ solution. To further analyze the effect of pore size on the transport rate, simulations of the transport of the NaCl + Na_2_SO_4_ solution in the γ–FeOOH/CSH nanopores were performed with four pore widths of 3.5 nm, 2.5 nm, 1.5 nm, and 1 nm.

**Figure 11:**
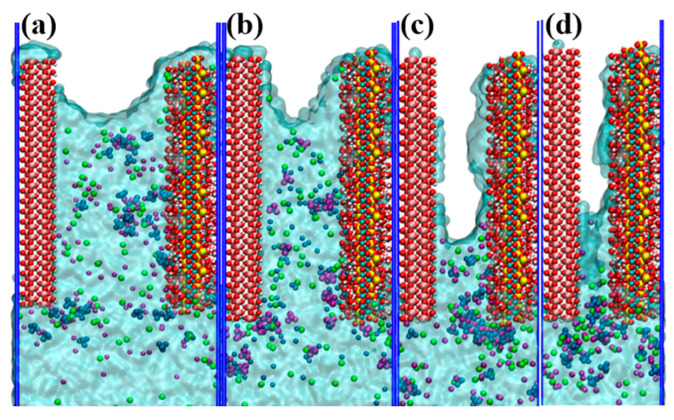
Snapshots of the capillary flow of the water molecules, Na^+^, Cl^−^, and SO_4_^2−^ ions in (**a**) 3.5 nm, (**b**) 2.5 nm, (**c**) 1.5 nm, and (**d**) 1.0 nm pores at 2000 ps. (Color codes for atoms were pink: Fe; yellow: Si; cyan: Ca; red: O; white: H; green: Cl; purple: Na; blue: SO_4_^2−^).

Snapshots of the capillary flow of the NaCl + Na_2_SO_4_ solution in these pores at *t* = 2000 ps are shown in Figure 11. As the pore width decreased from 3.5 nm to 1 nm, the penetration depths of the NaCl + Na_2_SO_4_ solution along both sides of the γ–FeOOH/CSH nanopore decreased. Furthermore, the penetration rate of all the ions, the Na^+^, Cl^−^, and SO_4_^2−^ ions, were smaller than that of the water molecules, and were further reduced with decreasing pore width. In the 1.5 nm and 1 nm pores, when the water molecules had migrated along the entire CSH surface, the ions had only migrated 30% to 40% of that surface. Pores of small width filtered water and ions effectively. This will be discussed in detail in the next subsection.

**Figure 12:**
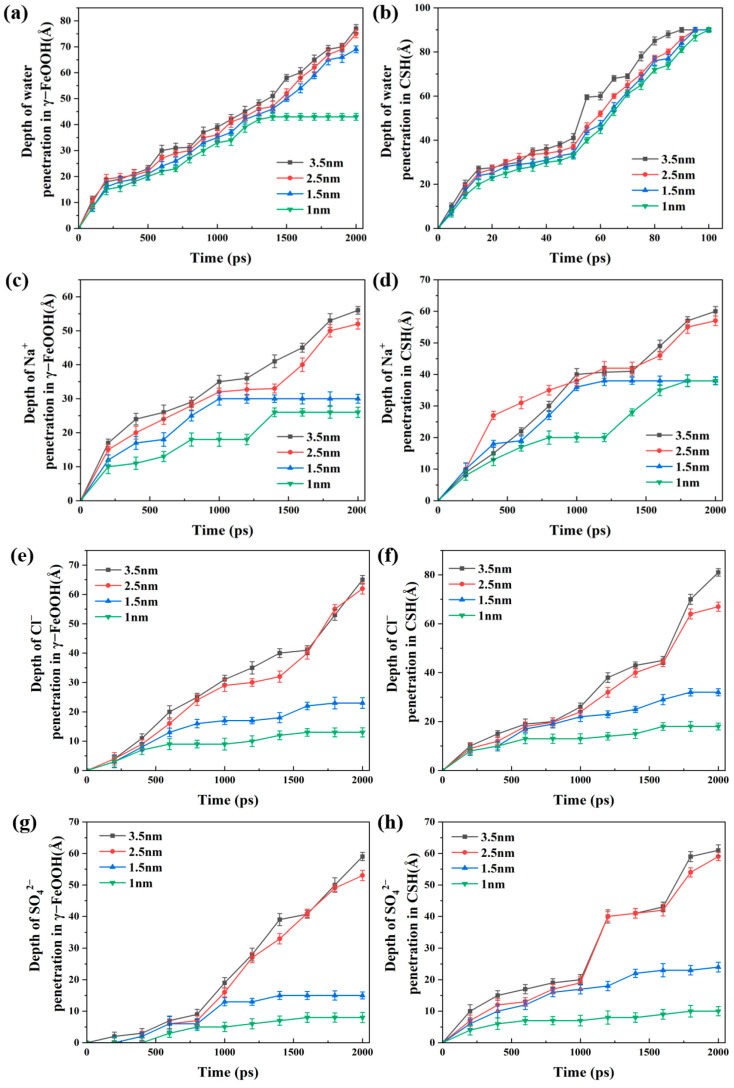
Penetration depths of (**a**) water, (**c**) Na^+^, (**e**) Cl^−^, and (**g**) SO_4_^2−^ along the γ–FeOOH surface and penetration depths of (**b**) water, (**d**) Na^+^, (**f**) Cl^−^, and (**h**) SO_4_^2−^ along the CSH surface at different pore sizes.

Further quantification of the migration of the NaCl + Na_2_SO_4_ solution in pores of different widths is given in Figure 12, where the positions of ions and water molecules on the substrates’ surfaces are plotted as functions of time. Comparing Figure 12a,b, it can be observed that the intrusion depth of water molecules on the surface of CSH was higher than that on γ–FeOOH, which is consistent with the results presented in Section 3.1. With the decrease in pore widths, the intrusion depths of water molecules on both substrate surfaces decrease. The “filtration” of water molecules was most evident for the 1 nm pore. At *t* = 1400 ps, the transport of water molecules on the γ–FeOOH was nearly halted. At 2000 ps, less than 50% of the γ–FeOOH surface was covered by the water.

Figure 12c–h depict the intrusion depths of the Na^+^, Cl^−^, and SO_4_^2−^ ions on both surfaces of pores with different widths. The intrusion depths of the three ions were smaller than that of the water molecules at any time, i.e., the migration of the ions was not synchronized with water. The curves in the 1.5 nm and 1 nm were lower than those in the 2.5 nm and 3.5 nm pores. At *t* = 2000 ps, the penetration depths of the Na^+^ ions on the γ–FeOOH surface were 5.6 nm, 5.2 nm, 3 nm, and 2.6 nm for the four pore widths, while those on the CSH surface were 6 nm, 5.7 nm, 3.8 nm, and 3.8 nm, respectively. This indicated that the blocking effect of the substrate surfaces on ion transport became stronger with decreasing pore size. The blocking effect was most pronounced for pore widths smaller than 2.5 nm. This observation also applied to the Cl^−^ and SO_4_^2−^. At *t* =1000 ps, the rate of penetration of the Cl^−^ and SO_4_^2−^ ions reduced, indicating that the blocking effect of small pore sizes on the Cl^−^ and SO_4_^2−^ ions was more pronounced than on the Na^+^ ions. The difference in the blocking effect of γ–FeOOH/CSH nanopore on ions and water molecules was mainly due to the differences between the interface of CSH(γ–FeOOH)/water and the interface C–S–H(γ–FeOOH)/ions.

The different transport rates of NaCl + Na_2_SO_4_ solution in different pore widths signified the strong effect of the width on the mobility of water and ions. Pores with smaller widths inhibited the penetration of water and ions, especially the diffusion of ions. The mechanism by which this happened required further investigation.

### 3.4. Local Structures of Water and Ions in Nano–Pores with Different Widths

**Figure 13:**
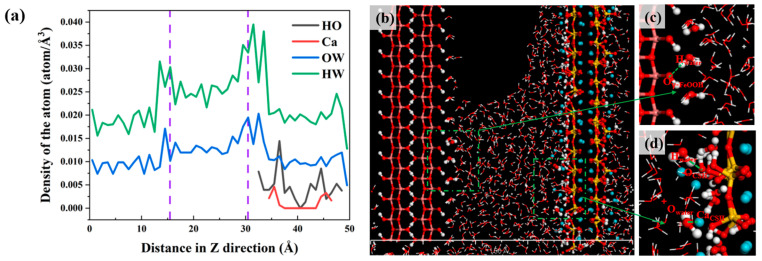
(**a**) Atomic density distribution perpendicular to the γ–FeOOH/CSH slit. Snapshot of the water molecule was (**b**) embedded in a 1.5 nm slit of γ–FeOOH/CSH and on the surface of (**c**) γ–FeOOH and (**d**) CSH. (Color codes for atoms were pink: Fe; yellow: Si; cyan: Ca; red: O; white: H; green: Cl; purple: Na; blue: SO_4_^2−^).

To understand the obstructing effect of pores with small widths on the solution transport, the density distribution of each constituent was calculated at *t* = 2000 ps. Figure 13a shows the density distributions of HO, Ca, O_w_, and H_w_ calculated with the 1.5 nm pore width.

Multiple peaks in the densities of the O_w_ and H_w_ atoms could be observed at and between the γ–FeOOH and CSH substrates, which indicated that the water molecules aggregated on the surface of γ–FeOOH and CSH and formed a layered structure. The distribution of H_w_ on the CSH surface had clear peaks at Z = 27.5, 29.5, 31.5, and 33.5 Å, with the largest peak at Z = 31.5 Å. On the γ–FeOOH surface, the density peaks of H_w_ were at Z = 13.5 Å and 15.5 Å. The density peaks of H_w_ and O_w_ on the γ–FeOOH surface were much smaller, which was explained by the stronger interaction between water molecules and the hydrophilic groups on the surface of CSH, including silicate chains and intra- and interlayer calcium ions. The first density peak of H_w_ was closer to the two substrates than that of O_w_. This is consistent with the results presented in Section 3.2 and indicates that the change in pore width does not affect the orientation distribution of the water molecules at the substrates. Figure 13b–d show that the water molecules were adsorbed on the substrates by forming hydrogen bonds with the hydroxyl groups in the outer layers of the CSH and γ–FeOOH. Furthermore, the water molecules also formed ionic bonds with the calcium ions on the surface of CSH. As a result, the hydroxyl hydrogen was always oriented towards the substrate, which indicated that the change in pore width does not affect the orientation distribution of water molecules. In summary, a series of changes in atomic density near the substrate were due to the hydrophilicity of γ–FeOOH and CSH and the formation of bonds with water molecules.

**Figure 14:**
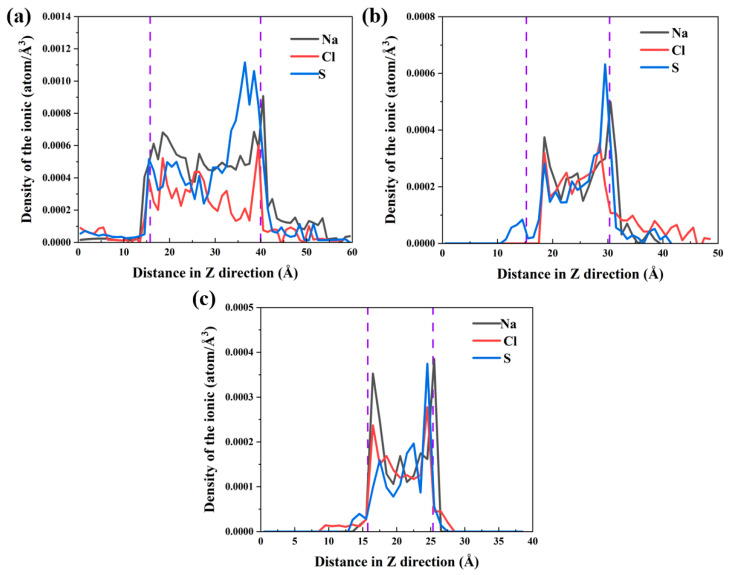
Density distribution of Na^+^, Cl^−^, and SO_4_^2−^ ions in the NaCl + Na_2_SO_4_ solution in (**a**) 2.5 nm, (**b**) 1.5 nm, and (**c**) 1 nm pores. (The left dashed line in the figure represents the surface of γ–FeOOH, while the right dashed line represents the surface of CSH).

To explain the blocking effect of smaller pore widths on the ions, the density distributions of the Na^+^, Cl^−^, and SO_4_^2−^ ions in the Z direction were calculated for the four pore widths and are shown in Figure 14.

Compared with the Cl^−^ and SO_4_^2−^ ions, the density peaks of the Na^+^ ions were closer to the surface of CSH and γ–FeOOH in the pores with four different widths. This shows that the change in pore width has no effect on the adsorption of the Na^+^ ions on the surfaces of CSH and γ–FeOOH, and the adsorption of cations on these surfaces is stronger than that of anions. All the ions were gathered on the surface of both substrates. However, fewer ions were accumulated on the γ–FeOOH surface compared to CSH; the density difference of the SO_4_^2−^ ions between the two substrates was most pronounced. This can be illustrated by the following example: In the pore with 2.5 nm width, the peak density of the SO_4_^2−^ ions on the CSH surface was up to 0.001 atom/Å^3^, whereas the peak density on the γ–FeOOH surface was only 0.0005 atom/Å^3^. When the pore width decreased, the SO_4_^2−^ ions entering the nanopore were mainly attracted by the Ca^2+^ ions on the CSH surface and attached to the CSH surface. On the contrary, the SO_4_^2−^ ions near the γ–FeOOH surface were not easily attracted. By comparing the density peaks of the same ion at different pore widths, it can be observed that the density peak of the ion decreased as the pore width decreased. In the four pore widths, the density peaks of Na^+^ ions on the CSH surface were 0.0011, 0.0009, 0.0005, and 0.0004 atom/Å^3^, respectively. These values represented 18%, 54%, and 63% reductions from those at 3.5 nm. The density peaks on the γ–FeOOH surface exhibited a similar trend, decreasing from 0.0007 atom/Å^3^ to 0.0006 atom/Å^3^, 0.0004 atom/Å^3^, and finally to 0.00035 atom/Å^3^ as the widths decreased. This indicated that the width reduction effectively blocked ions from entering the nanopores and impeded their transport. Notably, when the pore width was less than 2.5 nm, the ion density showed the most significant decrease, suggesting a more pronounced blocking effect.

**Figure 15:**
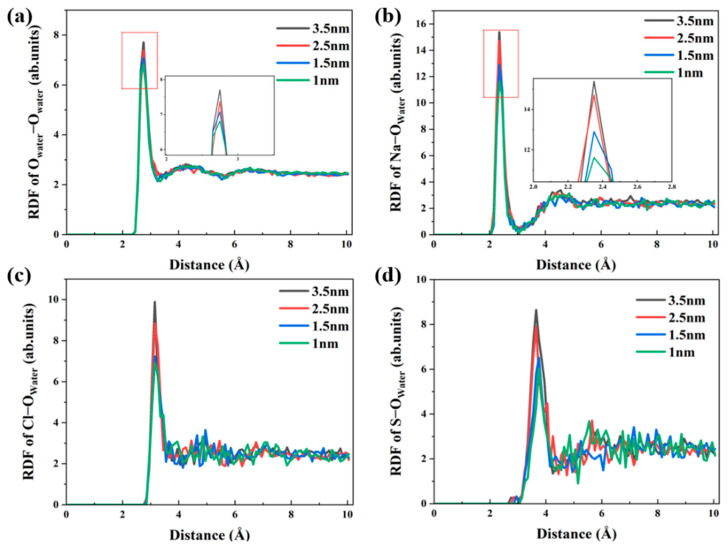
RDF of (**a**) O_water_–O_water_, (**b**) Na–O_water_, (**c**) Cl–O_water_, and (**d**) S–O_water_.

Further analysis of the pore width effect on the interaction between ions and water molecules was performed by studying the radial distribution functions (RDFs). The RDFs for water–water and water–ion interactions are shown in Figure 15. The first peak of the RDF for O_water_–O_water_ was observed at 2.75 Å, representing the closest interaction distance between water molecules. As pore width decreased, the peak diminished, indicating that smaller pore widths hindered water molecule interactions. The first peaks for Na–O_water_, Cl–O_water_, and S–O_water_ interactions in Figure 15b–d appeared at 2.35 Å, 3.15 Å, and 3.65 Å, respectively. Additionally, the Na–O_water_ RDF exhibited a second broadened peak at 4.45 Å, while Cl–O_water_ and S–O_water_ lacked significant secondary peaks. The peaks signified the radii of the first and the second hydration shells around each ion, and the hydration shell of Na–O_water_ had the smallest radius and the highest peak, suggesting stronger water–ion interactions. Decreasing pore width reduced the RDF peaks of Na–O_water_, Cl–O_water_, and S–O_water_, with the most pronounced differences occurring for widths smaller than 2.5 nm. These findings suggested that smaller widths weakened the water–ion attraction, leading to slower ion transport.

**Figure 16:**
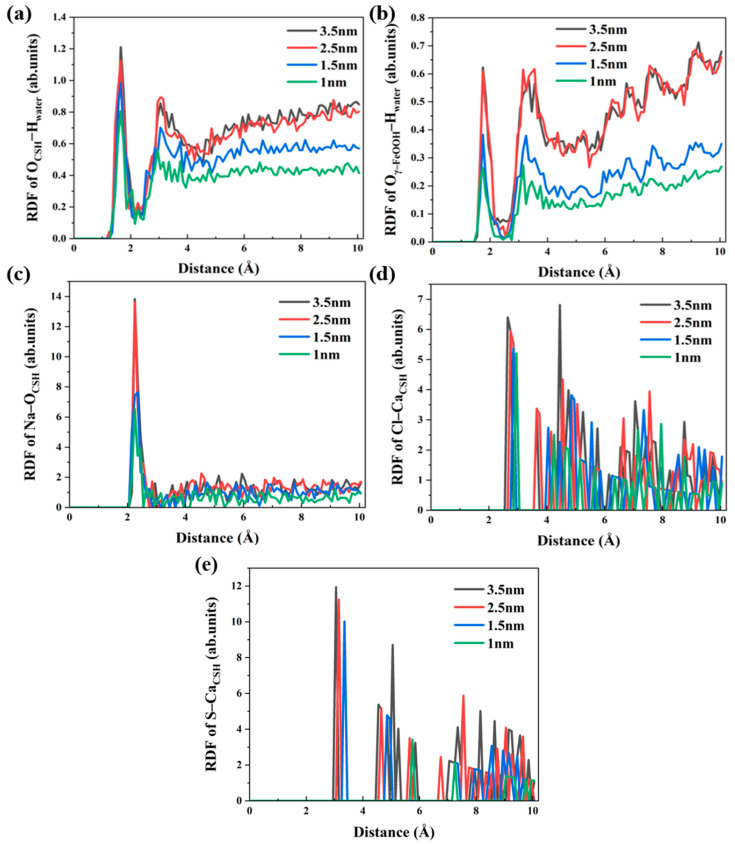
RDF of (**a**) O_CSH_–H_water_, (**b**) O_γ–FeOOH_–H_water_, (**c**) Na–O_CSH_, (**d**) Cl–Ca_CSH_, and (**e**) S–Ca_CSH_.

In addition to the interaction with solvated water molecules, aqueous species could form chemical bonds with CSH and γ–FeOOH. As depicted in Figure 16a,b, the RDFs for O_CSH_–H_water_ and O_γ–FeOOH_–H_water_ exhibited first peaks at 1.65 Å and 1.75 Å, respectively, indicating that hydrogen bonds formed between substrate surfaces and the adsorbed water molecules. With decreasing pore widths and diminishing peaks, the hydrogen bonds between the substrates and water molecules were weakened, which led to slower water molecules. The RDF peak of O_CSH_–H_water_ was much higher than that of O_γ–FeOOH_–H_water_ at the same pore width, indicating greater CSH affinity for water molecules compared to γ–FeOOH at all pore widths.

Figure 16c–e present RDFs for the Na^+^, Cl^−^, and SO_4_^2−^ ions interacting with the substrates at various pore widths. As pore width decreased, the RDF peaks of Na–O_CSH_, Cl–Ca_CSH_, and S–Ca_CSH_ decreased, suggesting weakened ion–CSH interactions. Their peaks were notably larger than those of O_CSH_–H_water_, indicating a stronger immobilization of ions by CSH. The hydroxyl groups on γ–FeOOH surfaces also adsorbed Na^+^ ions, forming Na–O_γ–FeOOH_ ion pairs. Reduced pore width decreased the distance between ion pairs on CSH surfaces and Na–O_γ–FeOOH_, promoting the formation of larger ion clusters such as O_γ–FeOOH_–Na–SO_4_–Ca and O_γ–FeOOH_–Na–Cl–Ca, which obstructed pore channels. Combining the RDF trends for ions and O_water_, it could be concluded that the reduction in the ion transport rate was due to weakened water molecule-driven ion motion. Moreover, the hydroxyl groups and Ca^2+^ ions on the CSH surface adsorbed the Na^+^, Cl^−^, and SO_4_^2−^ ions, retaining them for extended periods.

## 4. Conclusions

Molecular dynamics simulations were performed to clarify the transport mechanisms of water, NaCl, Na_2_SO_4_, and a composite solution of NaCl + Na_2_SO_4_ in γ–FeOOH/CSH nanopores. The effect of pore width on the transport of NaCl + Na_2_SO_4_ composite solution was also investigated. The following conclusions could be drawn:

(1) The transport of solutions in nanopores was meniscus-shaped with contact angles less than 90° on both substrates, which was consistent with capillary transport and confirms the hydrophilic nature of both CSH and γ–FeOOH.
(2) The penetration depths of different solutions were ordered as follows: D(water) > D(NaCl) > D(Na_2_SO_4_) > D(NaCl + Na_2_SO_4_). In all the solutions, water molecules penetrated along the CSH side faster than along the γ–FeOOH side. Ions penetrated more slowly than water molecules on both sides.
(3) Water molecules in the solution formed hydrogen bonds with γ–FeOOH and CSH, facilitating transport and driving ion movement. The nanopores immobilized different ions via distinct adsorption mechanisms: the Na^+^, Cl^−^, and SO_4_^2−^ ions formed Na–O_CSH_, Cl–Ca_CSH_, and S–Ca_CSH_ ion pairs with CSH, while Na–O_γ–FeOOH_ ion paired with γ–FeOOH. In the mixed solution of NaCl + Na_2_SO_4_, water and ion transport were inhibited by the formation of large clusters of ion pairs and their adsorption onto the CSH side.
(4) The transport rate of water and ions reduced with decreasing pore width, and the difference between the penetration depths of the water molecules and ions increased due to capillary action. For pores with less than 2.5 nm width, the Na^+^, Cl^−^, and SO_4_^2−^ ions tended to remain in the entry region of the nanopore.
(5) The Na–O_CSH_, Cl–Ca_CSH_, and S–Ca_CSH_ ion pairs formed by CSH with the Na^+^, Cl^−^, and SO_4_^2−^ ions were significantly stronger than the hydrogen bonds between CSH and water molecules. As a result, water transport continued while the ions were retained on the CSH surface for extended periods. This led to the separation of ions from water. For pores with less than 2.5 nm width, the proximity of the substrate surfaces caused the ion pairs adsorbed on the two surfaces to interact, forming a large cluster of ion pairs such as O_γ–FeOOH_–Na–SO_4_–Ca and O_γ–FeOOH_–Na–Cl–Ca. This partially blocked the pore entrance and hindered the transport of water molecules and ions.

In conclusion, this study focused on the transport process of γ–FeOOH/CSH interface nanopores under different erosion ions and different pore sizes, rather than solely the CSH nanopores, by MD simulation. The different mechanisms of solution invasion on the surfaces of two different matrices were revealed at the molecular level. Although the simulation results were limited to nanosecond and nanometer, the laws were applicable to engineering that the addition of steel fibers could effectively slow down the transmission of seawater in the concrete matrix, thereby enhancing the impermeability of port engineering structures. Furthermore, the idealized model was applied in a simulation as the initial exploration and research of this new type of material. More in-depth research on the durability of concrete is expected to be conducted, such as temperature, interface roughness, more comprehensive corrosive ions in seawater, and pore size effects. At present, steel fiber-reinforced concrete is widely used in port construction. It is believed that conducting research from a microscopic perspective will help us understand the working mechanism of this material in solutions, clarify the mechanism of enhancing the durability of SFRC, and hopefully provide support for better predicting its durability in the future.

## Figures and Tables

**Table 1:** Mix proportion of steel fiber-reinforced concrete (kg/m^3^).

Cement	Silica Fume	Fly Ash	Water	Superplasticizer	Steel Fiber	Quartz Sand	Coarse Aggregate
559.68	79.95	159.908	127.9264	15.9908	157	799.54	600

**Table 2:** Number of particles in solutions.

	H_2_O	Na^+^	Cl^−^	SO_4_^2−^
Water	5290	0	0	0
NaCl	5223	84	84	0
Na_2_SO_4_	5223	68	0	34
NaCl + Na_2_SO_4_	4948	152	84	34

## Data Availability

The original contributions presented in this study are included in the article. Further inquiries can be directed to the corresponding author.
